# Comparison of the Effects of Browning-Inducing Capsaicin on Two Murine Adipocyte Models

**DOI:** 10.3389/fphys.2019.01380

**Published:** 2019-11-05

**Authors:** Tommaso Montanari, Federico Boschi, Monica Colitti

**Affiliations:** ^1^Department of Agricultural, Food, Environmental and Animal Sciences, University of Udine, Udine, Italy; ^2^Department of Computer Science, University of Verona, Verona, Italy

**Keywords:** lipid droplets, 3T3-L1, X9, adipocytes, capsaicin, thermogenesis

## Abstract

The increasing prevalence of obesity and its associated comorbidities has gained attention in developing effective treatments and strategies that promote energy expenditure and the conversion of fat from a white to a brite phenotype. Capsaicin, bioactive component of chili peppers and a transient receptor potential channel vanilloid 1 (TRPV1) agonist, has been known to stimulate the process of thermogenesis. In this study, the effects of capsaicin were assessed on two murine cellular models by quantifying the dynamic of lipid droplets (LDs) and the expression of genes involved in adipocyte browning. Present findings demonstrated that treatment with norepinephrine or capsaicin combined with norepinephrine on 3T3-L1 cells and X9 cells significantly promoted the reduction of LDs area surface and size. The transcription of browning related genes such as uncoupling protein 1 (*Ucp1*), T-box transcription factor 1 (*Tbx1*), PR domain containing 16 (*Prdm16*), peroxisome proliferator-activated receptor γ coactivator 1α (*Ppargc1a*) and cell death-inducing DNA fragmentation factor A-like effector A (*Cidea*) was up-regulated by chronic capsaicin treatment on differentiated 3T3-L1 cells. Instead, X9 cells were significantly responsive only to the treatment with norepinephrine, used as positive control.

## Introduction

In recent years, research on adipose tissue biology and obesity has recognized in browning of white adipose tissue (WAT) a potential therapeutic strategy for the treatment of obesity and related morbidities (Peschechera and Eckel, 2013; Bartelt and Heeren, 2014). Unlike fat-accumulating WAT, brown adipose tissue (BAT) promotes the dissipation of energy deriving from the catabolism of fatty acids in form of heat. This process is known as thermogenesis and is driven by mitochondrial proton pump uncoupling protein 1 (UCP1) (Cannon and Nedergaard, 2004). The discovery of physiologically active BAT in adult humans (Cypess et al., 2009; Gifford et al., 2016) opened the investigation on therapeutic strategies for obesity based not only on the reduction of energy intake, but also on the increase of energy expenditure through the conversion of white adipocytes into brown-like ones, named beige or brite (brown-in-white) adipocytes (Wu et al., 2012).

The activation of BAT and the recruitment of brite adipocytes in WAT depots is mainly driven by cold exposure, which triggers a sympathetic response that induces the release of norepinephrine (NE) from sympathetic neurons and the activation of β_3_ adrenoreceptors on the membrane of adipocytes. This produces a signaling cascade involving protein kinase A (PKA) and p38 mitogen activated protein kinase (MAPK), which results in the expression of *Ucp1* and other key transcription factors involved in the activation of thermogenesis (Cannon and Nedergaard, 2004). Thermogenically active adipocytes show a different architecture of organelles than white fat cells. Brown and brite adipocytes accumulate triglycerides in a multilocular depot, hence their cytoplasm shows a high number of small lipid droplets (LDs) (Montanari and Colitti, 2018). This organization optimizes the process of lipolysis, in order to provide fatty acids promptly driven to fuel thermogenesis (Gao and Houtkooper, 2014). The dynamic of LDs in adipocytes is regulated by LD-linked proteins, with a role in LD enlargement and fusion. In murine BAT, members of cell death-inducing DNA fragmentation factor A (DFFA)-like effector (CIDE) protein family CIDEA and CIDEC are the main effectors of LD fusion. While CIDEC is expressed in both WAT and BAT and is crucial in generating the unilocular fat depot in white adipocytes (Nishimoto and Tamori, 2017), CIDEA is restricted to BAT, at least in mice, and it is not sufficient to induce the formation of supersized, white-like LDs by itself (Barneda and Christian, 2017).

Research on browning is currently focused in the individuation of both endogenous and exogenous molecules that could trigger the recruitment of thermogenically active brite adipocytes in WAT depots; up to date, a plethora of such molecules has been characterized, as largely reviewed (Montanari et al., 2017). Among these chemicals, natural compounds have gained a great interest, as they can be used as nutraceuticals to prepare functional foods with therapeutic properties.

Capsaicin belongs to a class of alkaloids, named capsaicinoids, found in the fruit of *Capsicum* spp. (hot pepper), which is responsible for the spicy flavor (Reyes-Escogido Mde et al., 2011). Its biological effects derive from the interaction with the transient receptor potential vanilloid 1 (TRPV1), a transmembrane receptor expressed mainly in peripheral sensory neurons involved in pain sensation (Bevan et al., 2014), widely distributed in the gastrointestinal tract (Yu et al., 2016), but also in adipose tissue (Bishnoi et al., 2013). The activation of TRPV1 by capsaicin opens the receptor’s ion channel, which allows the accumulation of intracellular Ca^2+^. Growing levels of Ca^2+^ activate the Ca^2+^/calmodulin-activated protein kinase (CaMKII), which in turn phosphorylates the cAMP-activated protein kinase (AMPK) and, finally, sirtuin 1 (SIRT1). The activated SIRT1 deacetylases the positive regulatory domain containing 16 (PRDM16) and peroxisome proliferator-activated receptor γ (PPARγ), which are key transcriptional factors actively involved in the positive regulation of *Ucp1* expression (Baskaran et al., 2016). In this regard, Ohyama et al. (2016) proposed that capsainoids signal, originating in the gut, is transmitted through central nervous system (CNS) to inguinal WAT by the β_*2*_-adrenoceptor.

Recent findings described the browning potential of capsaicin on white adipocytes (Baboota et al., 2014; Baskaran et al., 2016), but long-term effects on adipocytes still need further investigation. Moreover, the effect of capsaicin on LD size and number and on LD-associated proteins are still unclear and deserve deeper studies.

The present study enriches previous results on the browning potential of capsaicin with an investigation of the role of this nutraceutical on two different murine cellular models. These lines differ to their origin and could show different responses to browning stimulation, being X9 cells usually more prone to display a brite phenotype, since they were isolated from an inguinal fat pad depot (Wu et al., 2012). However, 3T3-L1 cells, widely used to evaluate the potential application of various compounds and nutrients in the treatment of obesity (Ruiz-Ojeda et al., 2016), were described to display features of multiple adipocytes lineages following appropriate stimulation (Morrison and McGee, 2015). The characteristic features of these models may lead to some unexpected variation, since 3T3-L1 cells act as a model of visceral adipocytes and X9 are subcutaneous. The changes in LD dynamics and gene expression profile after the administration of different doses of capsaicin have been analyzed between two times of differentiation. Finally, long-term effects of capsaicin, in combination with NE, are also tested.

## Materials and Methods

### Chemicals and Culture Media

Dulbecco’s modified Eagle medium (DMEM) enriched with 4.5 g/L D-glucose, 110 mg/L sodium pyruvate and 862 mg/L L-alanyl-L-glutamine (GlutaMAX^TM^), DMEM/F-12 (1:1) medium enriched with GlutaMAX^TM^, fetal bovine serum (FBS), penicillin/streptomycin solution and amphotericin B solution were purchased from Gibco by Life Technologies (Thermo Fisher Scientific, Inc., Waltham, MA, United States). Capsaicin and rosiglitazone were purchased from Cayman Chemical (Ann Arbor, MI, United States). Dipyrromethene boron difluoride (BODIPY) 493/503 dye, TRIzol reagent, PureLink^TM^ RNA Mini Kit and Platinum^TM^ SYBR^TM^ Green qPCR SuperMix-UDG kit for real time PCR were purchased from Invitrogen (Thermo Fisher Scientific, Inc., Waltham, MA, United States). ImProm-II^TM^ Reverse Transcription System was purchased from Promega (Madison, WI, United States). Normal goat (NG) serum was purchased by Vector Laboratories (Burlingame, CA, United States). 4,6-diamidin-2-phenylindole (DAPI)-containing mounting medium and Fluo-8 AM were purchased from Abcam (Cambridge, MA, United States). Hank’s balanced salt solution (HBSS) and 4-(2-hydroxyethyl)-1-piperazineethanesulfonic acid (HEPES) were purchased from Euroclone S.p.A. (Pero, Italy). All other chemicals used in the experiment and not listed above were purchased from Sigma-Aldrich (Darmstadt, Germany).

### Cell Culture and Treatment

Murine 3T3-L1 preadipocytes (ZenBio, Inc., Durham, NC, United States) and X9 preadipocytes (LGC Standards Srl, Sesto San Giovanni, Italy) were chosen for this comparative experiment.

Before starting the experiments, the growth of 3T3-L1 and X9 cells with media differently formulated was tested. On 3T3-L1 cells, the differentiation medium currently in use in our laboratory and inferred from literature was adopted. The insulin-IBMX-dexamethasone cocktail was so far the best solution to achieve excellent differentiation rates and white fat phenotype in 3T3-L1 cells. A growth protocol on X9 cells with the same growth conditions resulted in a poor differentiation rate, since quite 85% cells remained undifferentiated. The protocol suggested by Wu and Xu (2017) was then applied. Next, this medium for X9 was administered on 3T3-L1 cells, but the presence of rosiglitazone and triiodothyronine in the differentiation cocktail increased the *Ucp1* expression even if 3T3-L1 cells were not incubated with any browning factor (Chu et al., 2014). Since the administration of X9 medium could bias the results of *Ucp1* expression, different induction cocktails on two cellular models were applied as follows.

3T3-L1 preadipocytes were cultured in high-glucose DMEM supplemented with 10% FBS, 1% penicillin/streptomycin solution and 1% amphotericin B solution until 100% confluence. The differentiation was induced 48 h post-confluence by feeding cells with DMEM/F-12 supplemented with 10% FBS, 1% penicillin/streptomycin solution and 1% amphotericin B solution and enriched with 1 μg/mL insulin solution, 0.5 μM dexamethasone, and 0.5 mM isobutylmethylxanthine (IBMX) for 3 days. At day 3, maintenance was guaranteed by feeding cells with the differentiation basal medium supplemented with 1 μg/mL insulin solution; this medium was refreshed every 2 days.

X9 preadipocytes were grown in DMEM/F-12 supplemented with 15% FBS, 1% penicillin/streptomycin solution and 1% amphotericin B solution until 90% confluence. Differentiation was induced using the same differentiation basal medium formulated for 3T3-L1 cell culture enriched with 1 μg/mL insulin, 5 μM dexamethasone, 0.5 mM IBMX, 1 nM triiodothyronine (T_3_), and 1 μM rosiglitazone for 3 days. At day 3, cells were switched to the maintenance medium supplemented with 1 μg/mL insulin and 1 nM T_3_; the medium was refreshed every 2 days.

**FIGURE 1:**
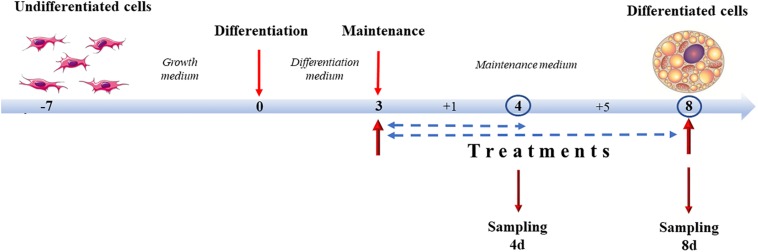
Cell culture treatment protocol. Cells were grown until 90% (X9) or 100% (3T3-L1) confluence. Differentiation lasted 3 days. From day 3, cells were incubated with maintenance medium supplemented with different treatments lasting for 1 (4d) or 5 days (8d) (sampling times).

Treatment of 3T3-L1 cells and X9 cells with capsaicin and NE, alone or in association, started at day 3 of differentiation, concurrently with the switch from the differentiation medium to the maintenance medium, and lasted until analysis either at 4 days of differentiation (4d), 1 day of treatment, or at 8 days of differentiation (8d), 5 days of treatment. Experimental groups were identified by adding to the maintenance medium different combinations of compounds. A vehicle negative control (CTRL) was established by adding to the maintenance medium absolute ethanol (1:1000 dilution). The positive control was represented by cells treated with 10 μM norepinephrine, the main endogenous browning molecule (NE). Capsaicin concentrations were tested at 0.1 μM (0.1CP) and 1 μM (1CP). To determine if any synergies/antagonisms between NE and capsaicin occurred and to mimic cold-independent adrenergic response that occurs *in vivo* after capsaicin stimulation (El Hadi et al., 2019), samples were also treated with both 10 μM norepinephrine and 0.1 μM capsaicin (0.1CPNE) or 1 μM of capsaicin (1CPNE) (Figure 1).

### Cell Viability Assay

Cell viability was determined by 3-(4,5-dimethylthiazol-2-yl)-2,5-diphenyltetrazolium bromide (MTT) assay. Cells were plated in a 96-well plate and treated with different concentrations of capsaicin and norepinephrine. Prior to incubation with 5 mg/mL MTT in HBSS, cells were rinsed with phosphate buffer saline (PBS) 1X. Incubation with MTT solution was performed at 37°C for 4 h and the resulting formazan was dissolved in dimethyl sulfoxide (DMSO) and incubated overnight (O/N) at 37°C. The optical density was used as an indicator of cell viability and was measured at 550 nm.

### BODIPY Staining and Confocal Imaging

Cells for BODIPY staining and subsequent confocal imaging were cultured on ibiTreat 8-well μ-Slides (Ibidi GmbH, Planegg/Martinsried, Germany). Cells were fixed in a 2% formalin solution diluted in PBS 1X at room temperature (RT) for 15 min. Subsequently, after three washes in PBS 1X, cells were incubated in a solution of BODIPY 493/503 in PBS 1X to fluorescently label lipid droplets. The incubation was performed at RT in dark for 45 min. After the incubation, the slides were washed in PBS 1X three times and then mounted with a DAPI-containing mounting medium. Fluorescent images were obtained using a Leica SP8 confocal microscope (Leica Microsystems Srl, Milan, Italy) equipped with LAS X 3.1.5.16308 software. Slides were observed with HC PL APO CS2 40X/1.10 WATER or HCX PL APO lambda blue 63X/1.40 OIL objective lenses. DAPI fluorescence was detected by diode 405 laser (410/480 nm), while the fluorescence of BODIPY was detected by white light laser (503/588 nm). The images were acquired by a photomultiplier tube (PMT), which allowed point-by-point scanning of the region of interest (ROI) with the selected laser and produced 1024 × 1024 px images.

### Morphology of LDs

MRI_Lipid Droplets tool^1^, a macro of ImageJ 1.50b software^2^, was used to measure LD area (Bäcker, 2012). The images were analyzed as already described (Colitti et al., 2018). For each LD, the area surface (in μm^2^), the maximum Feret diameter (MFD, in μm), and integrated optical density (IOD, dimensionless), were measured. The MDF is used as a measure of the diameter of irregularly shaped objects, while IOD is related to both triglyceride accumulation and LDs size (Boschi et al., 2019).

### RNA Extraction and RT-PCR

RNA extraction was performed by adding 1 mL/10 cm^2^ TRIzol reagent on the culture plate. The reagent was repeatedly pipetted to induce a severe breakdown of cells. The lysates in TRIzol were immediately processed with the PureLink^TM^ RNA Mini Kit following the manufacturer’s instructions. The concentration of the isolated total RNA was quantified using a Spark multimode microplate reader (Tecan Trading AG, Switzerland) and the purity of RNA samples was over 1.9. RNA integrity was evaluated through the observation of 18 and 28S ribosomal bands after electrophoresis on 1% agarose gel with GelRed. Primer3 Input software (Rozen and Skaletsky, 2000) was used to design primers. GenBank accession, primer sequences, product lengths, and relative annealing temperatures for each gene are listed in Table 1, according to the HUGO Gene Nomenclature Committee.

**TABLE 1:** Oligonucleotide primer sequences for Real-time PCR (*36b4*; RPLP0 ribosomal protein; *Gapdh*, glyceraldehyde-3-phosphate dehydrogenase; *Adrb3*, adrenoceptor beta 3; *Ucp1*, uncoupling protein 1; *Tbx1*, T-box 1; *Trpv1*, transient receptor potential vanilloid 1; *Prdm16*, proline rich domain containing 16; *Ppargc1a*, peroxisome proliferator-activated receptor γ coactivator 1 α; *Cidea*, cell death-inducing DFFA-like effector A; *Cidec*, cell death inducing DFFA like effector C; *Plin1*, perilipin 1).

**Gene**	**GenBank**	**Primer sets**	**Product**	**T_m_**
	**accession**		**length (bp)**	**(°C)**
*36b4*	BC099384.1	Forward: 5′-GAAACTGCTGCCTCACATCC-3′ Reverse: 5′-AGGTCTTCTCGGGTCCTAGA-3′	179	59.0
*Gapdh*	NM_008084	Forward: 5′-AATGTGTCCGTCGTGGATCTGA-3′ Reverse: 5′-AGTGTAGCCCAAGATGCCCTTC-3′	117	60.0
*Adrb3*	NM_013462.3	Forward: 5′-CCAATGACTCCTATGACC-3′ Reverse: 5′-TTCTGGAGCGTTGGAGAGTT-3′	89	57.3
*Ucp1*	NM_009463.3	Forward: 5′-CCCTGCCATTTACTGTCAGC-3′ Reverse: 5′-TCGGTCCTTCCTTGGGTGTAC-3′	157	59.0
*Tbx1*	NM_011532.2	Forward: 5′-AGGCGGAAGGAAGTGGTATT-3′ Reverse: 5′-TACCAGTATCTACACCGCCC-3′	118	58.4
*Trpv1*	AY445519.1	Forward: 5′-CGAGATAGGCATAGCACCCA-3′ Reverse: 5′-TGCTTCATGGTGTCCCTCAT-3′	130	58.4
*Prdm16*	NM_027504.3	Forward: 5′-CCACCAGCGAGGACTTCAC-3′ Reverse: 5′-GGAGGACTCTCGTAGCTCGAA-3′	107	61.4
*Ppargc1a*	NM_008904.2	Forward: 5′-TATGGAGTGACATAGAGTGTGCT-3′ Reverse: 5′-CTGGGCAAAGAGGCTGGTC-3′	191	60.0
*Cidea*	NM_007702.2	Forward: 5-ATCACAACTGGCCTGGTTACG-3′ Reverse: 5′-TACTACCCGGTGTCCATTTCT-3′	136	58.9
*Cidec*	NM_178373.4	Forward: 5′-ACCTTCGACCTGTACAAGCT-3′ Reverse: 5′-GTGCAGGTCATAGGAAAGCG-3′	99	58.4
*Plin1*	NM_175640.2	Forward: 5′-TGGACCACCTGGAGGAAAAG-3′ Reverse: 5′-CTTCGAAGGCGGGTAGAGATG-3′	94	60.6

*Tm = annealing temperature.*

Total RNA (500 ng) from each sample was reverse-transcribed with the ImProm-II^TM^ Reverse Transcription System in a MJ thermal cycler PT-100 (MJ Research, Inc., Waltham, MA, United States). For each gene, an aliquot of cDNA samples was pooled and standard curves with serial dilution of the pool were used to optimize real time PCR conditions in terms of cDNA and primers concentration and to calculate the efficiency, fluorescence baseline, and threshold.

Real time PCRs were performed for each sample in triplicate using Platinum^TM^ SYBR^TM^ Green qPCR SuperMix-UDG. PCR amplification was achieved applying 40 cycles (10 s at 95°C, 30 s at the specific annealing temperature, 30 s at 72°C) in a 96-well spectrofluorometric thermal cycler CFX (Bio-Rad, Milan, Italy). The melting curve analysis of amplification products was performed at the end of each PCR reaction to confirm that a single PCR product was detected.

The expression of target genes was normalized using the geometric means between RPLP0 ribosomal protein (*36b4*) and glyceraldehyde 3-phosphate dehydrogenase (*Gapdh*) mRNAs and analyzed using ΔΔCt method (Livak and Schmittgen, 2001). For all the cell culture experiments, the results are generated from biological triplicates and replicated similar results from at least three independent experiments.

### Immunofluorescence Analysis on 3T3-L1 Cells

Formalin-fixed cells were washed twice with PBS 1X containing 0.05% Tween-20 (PBST) at pH 7.4. Permeabilization of plasma membrane was performed by Triton X-100 0.1% incubation at RT. Fixative-induced autofluorescence was quenched with 50 mM ammonium chloride. Background labeling was prevented by incubating cells in a blocking solution containing 10% FBS and 5% NG serum in PBST 1X for 1 h at RT. The slides were then incubated overnight at 4°C in a moist chamber with the primary antibody diluted in blocking solution.

The anti-UCP1 polyclonal antibody (ab10983, Abcam, Cambridge, MA, United States) was raised against a peptide mapping to amino acids 145–159 of human UCP1 protein. The anti-PLIN1 was a rabbit polyclonal antibody (sc-67164, Santa Cruz Biotechnology, Inc., Heidelberg, Germany) raised against amino acids 1–300 mapping at the N-terminus of perilipin of human origin. Monoclonal anti-TRPV1 (sc-398417) was generated versus N-terminal amino acids 1–130 of rat TRPV1.

The following day, cells were incubated with 1:1000 AlexaFluor^®^ 555 goat anti-rabbit IgG (ab150078, Abcam, Cambridge, MA, United States), 1:200 fluorescein goat anti-rabbit IgG (FI-1000, Vector Laboratories, Burlingame, CA, United States) or 1:200 fluorescein goat anti-mouse IgM (FI-2020, Vector Laboratories, Burlingame, CA, United States) in blocking solution at RT for 45’ in the dark. Cells were then mounted with DAPI-containing mounting medium. Images were acquired with the fluorescence microscope Axio Observer Z1 equipped with D-PLAN Neofluar objective lenses with N.A. 0.75 and Infinity Color Corrected System (ICS) and with AxioCam and Zen blue software (Carl Zeiss, Jena, Germany). The filters were 550/605 nm for Alexa Fluor^®^ 555 antibody conjugate, 470/525 nm for fluorescein antibodies and 390/460 nm for DAPI.

### Intracellular Calcium Analysis on 3T3-L1 Cells

Differentiated cells at 4 and 8 days cultured on ibiTreat 8-well μ-Slides were incubated, after culture medium removal, with a 4 μM Fluo-8 AM working solution in HBSS supplemented with 15 mM HEPES for 1 h at 37°C. Stained cells were then rinsed with HEPES-HBSS and immediately imaged at Axio Observer Z1 fluorescence microscope with the 470/525 nm filter. The administration of 1 μM capsaicin was performed directly on the slide during the movie acquisition, 12–14 s after the beginning of the movie. Each acquisition lasted 180 s.

Analysis intensity over time was analyzed within selected ROIs with ImageJ^3^ and the influx of Ca^2+^ was quantified, after background subtraction, as the ratio between the maximum fluorescence intensity emitted after the addition of the compound (F) and the average of baseline fluorescence (F0), recorded before the treatment. To quantify the ON-OFF response of 3T3-L1 adipocytes, standard deviation of the fluorescence intensity was calculated using ImageJ (Muto et al., 2013).

### Statistical Analysis

Results are presented as relative values (means ± SD). All experiments were performed at least three times. One-way ANOVA was used for statistical analysis for MTT test by SPSS version 20.0 software.

The measurements of total cell area surface, MFD and IOD were analyzed by XLSTAT (Addinsoft, 2017). Results, obtained from 10 biological replicates, were compared using through Kruskal–Wallis statistical test followed by pairwise comparisons using the Mann–Whitney approach with Bonferroni correction.

Gene expression data (relative fold change values) were analyzed within the same cell line by one-way ANOVA analysis using XLSTAT with Bonferroni correction (*p* = 0.003) and reported as least squares means (LS) ± standard error of the mean (SEM).

The relative fold change values were subjected to enrichement analysis of biological process (BP), performed by Funrich tool 3.1.3 against mouse UniProt database. Bonferroni test was applied to correct for multiple testing (Pathan et al., 2015).

Principal component analysis (PCA) was used to perform multivariate analysis of correlated variables between the percentage of protein annotation obtained by relative fold change uploaded on Funrich and the significant (*p* < 0.05) BP, at 4 and 8 days, respectively. PCA simplifies the data complexity while retaining trends and patterns. Biplots were drawn to have simultaneous representation of variables and observations in the PCA space.

## Results

### Cell Viability

The percentage of cell viability after treatment with NE, 0.1CP, 0.1CPNE, 1CP, 1CPNE at 4 and 8 days was always around 100% (Supplementary Figure S1). No significant differences were observed for time point and for concentration × time point interaction.

### Lipid Droplet Measurements and Analysis in 3T3-L1 Cells

**FIGURE 2:**
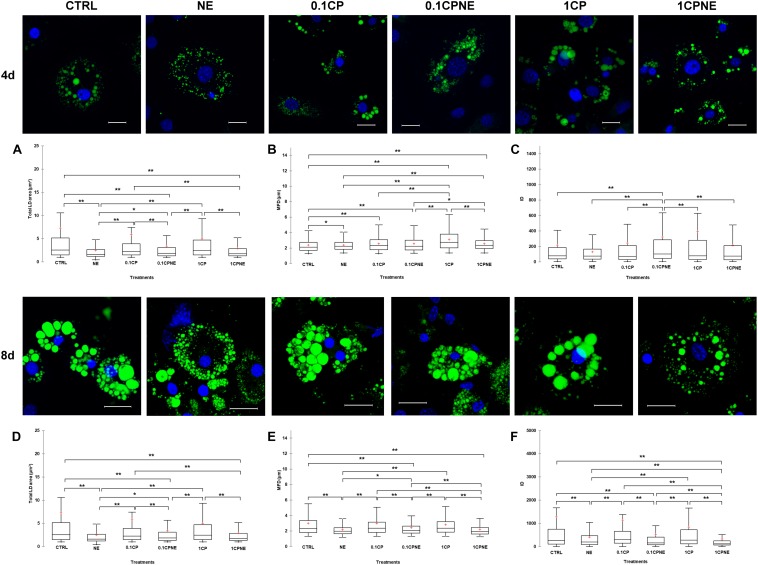
Lipid droplets formation stained with BODIPY 493/503 in fixed 3T3-L1 cells at 4 and 8 days of differentiation. Nuclear staining DAPI. Images are representative of n. 5 biological replicates. **(A–F)** Lipid droplets box plots show median (horizontal lines), first to third quartile (box), and the most extreme values with the interquartile range (vertical lines). For all comparison, differences between treatments on 3T3-L1 cells were statistically significant, using Kruskal–Wallis test. **(A)** Total LD area surface at 4 days; **(B)** Maximum Feret diameter (MFD) at 4 days; **(C)** Intensity optical density (IOD) at 4 days. **(D)** Total LD area surface at 8 days; **(E)** Maximum Feret diameter (MFD) at 8 days; **(F)** Intensity optical density (IOD) at 8 days. The area surface measures are expressed in μm^2^ and MFD μm. Measures are the average of 1224 and 853 observations at 4 at 8 days, respectively. ^∗∗^*p* < 0.0001; ^∗^*p* < 0.05. Scale bars: 20 μm. CTRL = vehicle negative control; NE = 10 μM norepinephrine; 0.1CP = 0.1 μM capsaicin; 0.1CPNE = 0.1 μM capsaicin plus 10 μM norepinephrine; 1CP = 1 μM capsaicin; 1CPNE = 1 μM capsaicin plus 10 μM norepinephrine.

Figure 2 illustrates LDs dynamic in 3T3-L1 cells at 4 and 8 days treated with different doses of compound. Tables showing mean values ± SD and *p*-values of total area surface, MFD and IOD among treatments at 4 and 8 days are reported in Supplementary Tables S1, S2.

Interestingly, at 4 days, 3T3-L1 cells treated with NE, 0.1CPNE and 1CP showed significantly (*p* < 0.0001) different total LDs area surface in comparison to CTRL cells and all other treatments (Figure 2A). 1CP showed the largest LD area surface and NE the smallest.

The MFD of 1CP-treated cells was significantly (*p* < 0.0001) higher with respect to all other treatments. Conversely, MFD of CTRL cells was the significantly smallest compared to other treatments (Figure 2B). MFD of NE cells differed significantly from CTRL ones (*p* < 0.05) and it is significantly (*p* < 0.0001) lower compared to 1CP and 1CPNE treatments (Figure 2B).

Figure 2C evidences statistically significant differences of IOD at 4 days. For all the treatments, the IOD of 0.1CPNE-treated cells was significantly different (*p* < 0.0001) to all treatments.

At 8 days a significant decrease in total area surface of LDs was observed in NE, which was significantly different from all treatments with the exception of 1CPNE (Figure 2D).

Maximum Feret diameter of 0.1CPNE-treated cells was significantly different among samples (Figure 2E).

The strongest (*p* < 0.0001) decrease in IOD value was observed in 1CPNE-treated cells followed by NE and 0.1CPNE (Figure 2F).

Taken together, the results obtained at 4 and 8 days with NE and 1CPNE treatments on 3T3-L1 cells showed a strongest effect in reducing LDs area surface and IOD, whereas 0.1CP and 1CP treatments were less active and similar to CTRL at 8 days.

### Lipid Droplet Measurements and Analysis in X9 Cells

**FIGURE 3:**
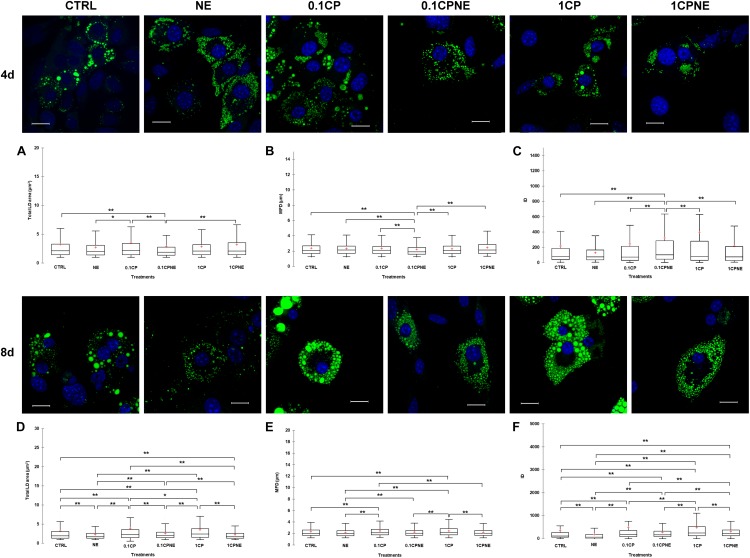
Lipid droplets formation stained with BODIPY 493/503 in fixed X9 cells at 4 and 8 days of differentiation. Nuclear staining DAPI. Images are representative of n. 5 biological replicates. **(A–F)** Lipid droplets box plots show median (horizontal lines), first to third quartile (box), and the most extreme values with the interquartile range (vertical lines). For all comparison, differences between treatments on X9 cells were statistically significant, using Kruskal–Wallis test. **(A)** Total LD area surface at 4 days; **(B)** Maximum Feret diameter (MFD) at 4 days; **(C)** Intensity optical density (IOD) at 4 days. **(D)** Total LD area surface at 8 days; **(E)** Maximum Feret diameter (MFD) at 8 days; **(F)** Intensity optical density (IOD) at 8 days. The area surface measures are expressed in μm^2^ and MFD μm. Measures are the average of 1824 and 1778 observations at 4 at 8 days, respectively. ^∗∗^*p* < 0.0001; ^∗^*p* < 0.05. Scale bars: 20 μm. CTRL = vehicle negative control; NE = 10 μM norepinephrine; 0.1CP = 0.1 μM capsaicin; 0.1CPNE = 0.1 μM capsaicin plus 10 μM norepinephrine; 1CP = 1 μM capsaicin; 1CPNE = 1 μM capsaicin plus 10 μM norepinephrine.

Figure 3 illustrates LDs dynamic in X9 cells at 4 and 8 days treated with different doses of compound. Tables showing mean values ± SD and *p*-values of total area surface, MFD and IOD among treatments at 4 and 8 days are reported in Supplementary Tables S3, S4.

After 4 days of development 0.1CPNE treatment showed the strongest effect on the measure of total area surface, particularly in comparison to CTRL, 0.1CP and 1CPNE (*p* < 0.0001). NE-treated cells were different (*p* < 0.05) to 0.1CP (Figure 3A).

Maximum Feret diameter values reflected the same trend, as 0.1CPNE showed the significant lowest value in analogy to total area surface (Figure 3B).

Integrated optical density value in cells treated with NE alone, 0.1CP and 1CP was significantly lower in comparison to other dosages, but again 0.1CPNE-treated cells were significantly (*p* < 0.0001) different to all other treated cells (Figure 3C).

At 8 days, all treatments significantly affected total LDs area surface, with 0.1CP and 1CP producing the largest surface and NE and 1CPNE the smallest one. No differences were observed between CTRL and 0.1CPNE cells (Figure 3D).

Maximum Feret diameter significantly increased in X9 cell treated with the two capsaicin concentrations (0.1CP and 1CP, *p* < 0.0001), whereas treatments coupled with NE negatively (*p* < 0.0001) affected the MFD size (Figure 3E).

Integrated optical density values were significantly different among all treatments and NE-treated cells showed the lowest value (*p* < 0.0001) (Figure 3F).

Results obtained on X9 cells at 4 and 8 days with NE and NE in combination with 0.1CP or 1CP treatments showed the strongest effect in reducing LDs area surface, MFD and IOD, whereas 0.1CP treatment was the least effective at both differentiation times.

### Comparison of LD Measurements Between Treatments at 4 and 8 Days in 3T3-L1 and in X9 Cells

**FIGURE 4:**
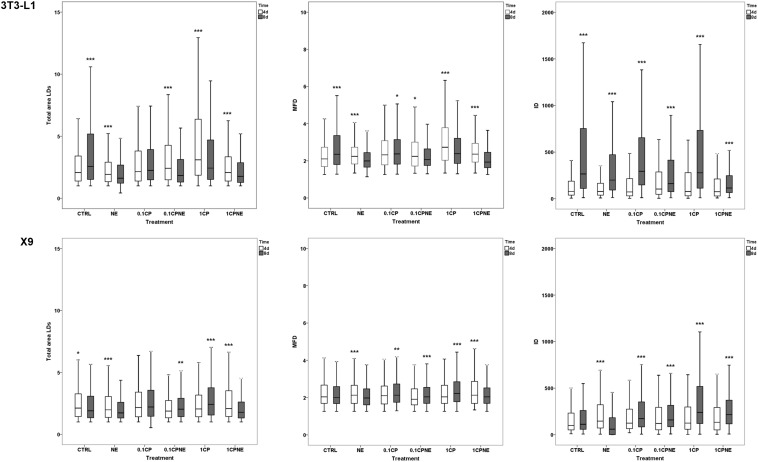
Pairwise comparison of total area surface, MFD and IOD between the same treatment after 4 (4d) and 8 days (8d) of differentiation within 3T3-L1 cells and X9 cells. Box plots show median (horizontal lines), first to third quartile (box), and the most extreme values with the interquartile range (vertical lines). For all comparison, differences between days of differentiation of 3T3-L1 and X9 cells were statistically significant using Kruskal–Wallis test. The measurements are the average of 10 fields of each developmental stage. ^∗∗∗^*p* < 0.0001, ^∗∗^*p* < 0.01, ^∗^*p* < 0.05. CTRL = vehicle negative control; NE = 10 μM norepinephrine; 0.1CP = 0.1 μM capsaicin; 0.1CPNE = 0.1 μM capsaicin plus 10 μM norepinephrine; 1CP = 1 μM capsaicin; 1CPNE = 1 μM capsaicin plus 10 μM norepinephrine.

Figure 4 illustrates the pairwise comparison of total area surface, MFD and IOD between the same treatment after 4 and 8 days of differentiation within 3T3-L1 cells and X9 cells.

Total LDs area surface in 3T3-L1 exhibited significant differences between CTRL cells being higher (*p* < 0.0001) at 8 days in comparison to 4 days. However, cells treated with NE, 0.1CPNE, 1CP, and 1CPNE showed the highest (*p* < 0.0001) area surface at 4 days.

Maximum Feret diameter was at its highest at 8 days in control cells (*p* < 0.0001) and in 0.1CP (*p* < 0.05), but significantly (*p* < 0.0001) decreased in NE, 1CP, 1CPNE-treated cells and in 0.1CPNE (*p* < 0.05).

Interestingly, IOD value was significantly (*p* < 0.0001) higher at 8 days in 3T3-L1 CTRL cells and in all treated cells.

Total LDs area surface in X9 cells was significantly higher at 4 days in CTRL cells (*p* < 0.05), NE and 1CPNE-treated cells (*p* < 0.0001), but significantly increased at 8 days in comparison to 4 days in 0.1CPNE (*p* < 0.01) and 1CP (*p* < 0.0001) treated cells.

Maximum Feret diameter size was significantly higher (*p* < 0.0001) in NE and 0.1CPNE X9 cells at 8 days, while 0.1CP (*p* < 0.05), 0.1CPNE and 1CPNE treatments (*p* < 0.0001) increased the value of MFD at 4 days in comparison to 8 days.

Control cells did not show any significant difference of IOD value between 4 and 8 days, only NE-treated cells decreased significantly (*p* < 0.0001) at 8 days, while other treatments showed an increase (*p* < 0.0001) at 8 days.

### Transcription Data Analysis

Table 2 showed the LS means of relative fold change values ± SEM of 3T3-L1 at 4 and 8 days.

**TABLE 2:** Differentially expressed genes in 3T3-L1 cells at 4 and 8 days.

**Treatment 4d**	***Adrb3***		**SEM**	***Ucp1***		**SEM**	***Tbx1***		**SEM**	***Trpv1***		**SEM**	***Prdm16***		**SEM**	***Ppargc1a***		**SEM**	***Cidec***		**SEM**	***Cidea***		**SEM**	***Plin1***		**SEM**
1CPNE	0.50	±	0.16	1.83	±	0.26	1.14	±	0.26	1.54	±	0.26	0.81^AB^	±	0.18	0.60	±	0.14	1.91	±	0.30	1.39^AB^	±	0.34	1.29	±	0.17
0.1CPNE	0.48	±	0.16	1.66	±	0.26	0.92	±	0.26	1.12	±	0.26	1.67^A^	±	0.18	0.38	±	0.14	0.66	±	0.30	2.94^A^	±	0.34	1.07	±	0.17
CTRL	1.04	±	0.16	0.90	±	0.26	1.00	±	0.26	1.00	±	0.26	1.01^AB^	±	0.18	1.01	±	0.14	0.79	±	0.30	0.89^B^	±	0.34	1.00	±	0.17
0.1CP	1.02	±	0.16	0.85	±	0.26	0.67	±	0.26	0.95	±	0.26	0.72^B^	±	0.18	0.82	±	0.14	1.36	±	0.30	0.89^B^	±	0.34	1.27	±	0.17
1CP	0.59	±	0.16	1.36	±	0.26	0.62	±	0.26	1.28	±	0.26	0.41^B^	±	0.18	0.67	±	0.14	0.84	±	0.30	1.14^AB^	±	0.34	1.05	±	0.17
NE	0.42	±	0.16	0.93	±	0.26	0.60	±	0.26	0.70	±	0.25	0.49^B^	±	0.18	0.34	±	0.14	0.72	±	0.29	1.27^AB^	±	0.34	0.78	±	0.17
Pr > F (Model)	0.060			0.091			0.105			0.343			0.004			0.042			0.090			0.023			0.367		

**Treatment 8d**	***Adrb3***		**SEM**	***Ucp1***		**SEM**	***Tbx1***		**SEM**	***Trpv1***		**SEM**	***Prdm16***		**SEM**	***Ppargc1a***		**SEM**	***Cidec***		**SEM**	***Cidea***		**SEM**	***Plin1***		**SEM**

1CP	2.71^A^	±	0.19	13.98^A^	±	0.63	21.47^A^	±	1.07	12.74^A^	±	0.67	16.45^A^	±	1.40	4.46^A^	±	0.47	4.37^A^	±	0.33	14.67^A^	±	0.71	3.20^A^	±	0.17
0.1CP	1.25^B^	±	0.16	1.96^B^	±	0.51	2.30^B^	±	0.87	2.47^B^	±	0.54	2.23^B^	±	1.14	1.43^B^	±	0.39	1.01^B^	±	0.27	3.25^B^	±	0.58	1.18^B^	±	0.14
0.1CPNE	0.45^BC^	±	0.16	2.92^B^	±	0.51	3.54^B^	±	0.87	2.80^B^	±	0.54	3.51^B^	±	1.14	0.68^B^	±	0.39	1.02^B^	±	0.27	1.52^B^	±	0.58	0.75^B^	±	0.14
1CPNE	0.34^C^	±	0.16	1.64^B^	±	0.51	1.61^B^	±	0.87	1.43^B^	±	0.54	1.47^B^	±	1.14	0.62^B^	±	0.39	0.98^B^	±	0.27	1.45^B^	±	0.58	0.61^B^	±	0.14
CTRL	0.99^BC^	±	0.16	0.85^B^	±	0.51	0.83^B^	±	0.87	0.89^B^	±	0.54	0.76^B^	±	1.14	1.02^B^	±	0.39	1.00^B^	±	0.27	0.82^B^	±	0.58	1.04^B^	±	0.14
NE	0.23^C^	±	0.19	1.15^B^	±	0.61	1.28^B^	±	1.05	0.94^B^	±	0.65	0.97^B^	±	1.36	0.41^B^	±	0.46	0.59^B^	±	0.32	0.79^B^	±	0.70	0.71^B^	±	0.17
Pr > F (Model)	<0.0001			< 0.0001			<0.0001			< 0.0001			0.001			0.001			<0.0001			< 0.0001			<0.0001		

*CTRL = vehicle negative control; NE = 10 μM norepinephrine; 0.1CP = 0.1 μM capsaicin; 0.1CPNE = 0.1 μM capsaicin plus 10 μM norepinephrine; 1CP = 1 μM capsaicin; 1CPNE = 1 μM capsaicin plus 10 μM norepinephrine. *Adrb3*, adrenoceptor beta 3; *Ucp1*, uncoupling protein 1; *Tbx1*, T-box 1; *Trpv1*, transient receptor potential vanilloid 1; *Prdm16*, proline rich domain containing 16; *Ppargc1a*, peroxisome proliferator-activated receptor γ coactivator 1 α; *Cidec*, cell death inducing DFFA like effector C; *Cidea*, cell death-inducing DFFA-like effector A; *Plin1*, perilipin 1. 4d = at 4 days of differentiation; 8d = at 8 days of differentiation. The data are shown as Least squares (LS) means ± standard error of the mean (SEM). Pr > F (Model) = *p*-value for the effect of the model on the response. Capital letters indicate a significant Bonferroni-corrected difference (*p* < 0.003).*

The treatment of 0.1CPNE significantly affected the expression of *Prdm16* and *Cidea* at 4 days. In particular, the expression of *Prdm16* induced by 0.1CPNE treatment significantly differed from 1CP (*p* = 0.00038), NE (*p* = 0.001), and 0.1CP (*p* = 0.003). *Cidea* expression was affected by 0.1CPNE and significantly differed from 0.1CP treatment (*p* = 0.003) and negative CTRL (*p* = 0.003). Bonferroni’s corrected significance level (*p* = 0.003) produced by the analysis penalized the significance (*p* = 0.042) of *Ppargc1a* expression among treatments. 1CPNE treatment most affected the gene expression and the effect of NE was the lowest on 3T3-L1 at 4 days.

The treatment of 1CP at 8 days in 3T3-L1 significantly (*p* < 0.0001) up-regulated the expression of all genes.

NE treatment did not produce any significant modulation of genes related to browning even at 8 days.

The LS means of relative fold change values ± SEM of X9 cells is presented for all the treatments at 4 and 8 days in Table 3.

**TABLE 3:** Differentially expressed genes in X9 cells at 4 and 8 days.

**Treatment 4d**	***Adrb3***		**SEM**	***Ucp1***		**SEM**	***Tbx1***		**SEM**	***Trpv1***		**SEM**	***Prdm16***		**SEM**	***Ppargc1a***		**SEM**	***Cidec***		**SEM**	***Cidea***		**SEM**	***Plin1***		**SEM**
NE	8.70^A^	±	1.24	3.34^A^	±	0.45	2.10	±	0.33	1.57	±	0.39	3.04	±	0.64	2.16^AB^	±	0.50	1.90	±	0.28	1.34	±	0.22	1.64	±	0.35
1CP	0.66^B^	±	1.25	0.94^AB^	±	0.45	0.92	±	0.33	1.16	±	0.40	0.95	±	0.65	4.07^A^	±	0.51	1.38	±	0.28	1.12	±	0.23	0.22	±	0.36
0.1CPNE	3.04^AB^	±	1.53	2.12^AB^	±	0.55	0.81	±	0.41	1.28	±	0.49	1.66	±	0.79	1.86^AB^	±	0.62	0.56	±	0.35	0.87	±	0.28	0.08	±	0.44
CTRL	0.96^B^	±	1.25	0.95^AB^	±	0.45	1.03	±	0.33	1.15	±	0.40	1.04	±	0.65	1.18^B^	±	0.51	1.00	±	0.28	1.13	±	0.23	1.15	±	0.36
0.1CP	0.64^B^	±	1.25	0.52^B^	±	0.45	0.71	±	0.33	0.73	±	0.40	0.84	±	0.65	2.79^AB^	±	0.51	1.25	±	0.28	1.37	±	0.23	0.42	±	0.36
1CPNE	2.44^AB^	±	1.25	0.93^AB^	±	0.45	0.69	±	0.33	1.12	±	0.40	2.69	±	0.65	1.68^AB^	±	0.51	0.44	±	0.28	0.74	±	0.23	0.09	±	0.36
Pr > F (Model)	0.007			0.018			0.088			0.790			0.136			0.029			0.039			0.383			0.064		

**Treatment 8d**	***Adrb3***		**SEM**	***Ucp1***		**SEM**	***Tbx1***		**SEM**	***Trpv1***		**SEM**	***Prdm16***		**SEM**	***Ppargc1a***		**SEM**	***Cidec***		**SEM**	***Cidea***		**SEM**	***Plin1***		**SEM**

CTRL	1.00^A^	±	0.07	0.66^B^	±	0.52	1.05	±	0.12	1.06	±	0.26	0.78^B^	±	0.33	1.02	±	0.14	1.02^ABC^	±	0.17	1.03^AB^	±	0.18	0.92^A^	±	0.13
NE	0.16^B^	±	0.07	3.59^A^	±	0.50	0.92	±	0.12	1.62	±	0.26	2.83^A^	±	0.33	0.95	±	0.14	0.60^BC^	±	0.17	0.50^B^	±	0.18	0.35^AB^	±	0.12
1CP	0.78^A^	±	0.07	1.11^AB^	±	0.52	0.72	±	0.12	0.99	±	0.26	0.82^B^	±	0.33	0.76	±	0.14	1.32^AB^	±	0.17	0.57^B^	±	0.18	0.66^AB^	±	0.13
0.1CP	0.96^A^	±	0.07	0.66^B^	±	0.52	0.58	±	0.12	0.74	±	0.26	0.56^B^	±	0.33	0.90	±	0.14	1.87^A^	±	0.17	1.89^A^	±	0.18	0.85^A^	±	0.13
0.1CPNE	0.14^B^	±	0.07	1.60^AB^	±	0.52	0.60	±	0.12	0.74	±	0.26	0.84^B^	±	0.33	0.44	±	0.14	1.04^ABC^	±	0.17	0.27^B^	±	0.18	0.37^AB^	±	0.13
1CPNE	0.09^B^	±	0.07	2.15^AB^	±	0.52	0.58	±	0.12	1.52	±	0.26	2.09^AB^	±	0.33	0.58	±	0.14	0.24^C^	±	0.17	0.12^B^	±	0.18	0.10^B^	±	0.13
Pr > F (Model)	<0.0001			0.015			0.072			0.125			0.002			0.088			0.000			0.000			0.005		

*CTRL = vehicle negative control; NE = 10 μM norepinephrine; 0.1CP = 0.1 μM capsaicin; 0.1CPNE = 0.1 μM capsaicin plus 10 μM norepinephrine; 1CP = 1 μM capsaicin; 1CPNE = 1 μM capsaicin plus 10 μM norepinephrine. *Adrb3*, adrenoceptor beta 3; *Ucp1*, uncoupling protein 1; *Tbx1*, T-box 1;*Trpv1*, transient receptor potential vanilloid 1; *Prdm16*, proline rich domain containing 16; *Ppargc1a*, peroxisome proliferator-activated receptor γ coactivator 1 α; *Cidec*, cell death inducing DFFA like effector C; *Cidea*, cell death-inducing DFFA-like effector A; *Plin1*, perilipin 1. 4d = at 4 days of differentiation; 8d = at 8 days of differentiation. The data are shown as Least squares (LS) means ± standard error of the mean (SEM). Pr > F (Model) = *p*-value for the effect of the model on the response. Capital letters indicate a significant Bonferroni-corrected difference (*p* < 0.003).*

*Adrb3*, *Ucp1*, *Cidec* expression was significantly modulated in X9 cells treated with NE at 4 days. Bonferroni’s corrected significance level (*p* = 0.003) produced by the analysis penalized the significance (*p* = 0.039) of *Cidec* expression among treatments. Notably, treatments coupled with NE significantly modulated gene expression in comparison to capsaicin treatments. *Ucp1* and *Prdm16* expression were significantly induced at 8 days by NE. Interestingly, no treatments induced a significant regulation of *Trpv1*.

### Biological Process Enrichment Analysis

The comparison of the relative fold change values among treatments and times were enriched in biological process (BP) using Funrich 3.1.3 finding tool. *p*-Values, after Bonferroni correction, were calculated based on the comparison within samples of 3T3-L1 cells at 4 and 8 days and of X9 at the same times. Funrich tool returned results as percentage of annotated proteins and the analysis produced 233 BPs. Among these, 29 were significant for *p* < 0.05. Within the significant BPs, the eight most enriched were chosen due to their relation with the study. All selected significant BPs showing the percentage of expression in 3T3-L1 and X9 cell, according to treatments and times, are reported in Supplementary Table S5. The most significant BP were “diet induced thermogenesis” (*p* < 2.7 × 10^–10^) and “brown fat cell differentiation” (*p* < 7.3 × 10^–9^) followed by “response to cold” (*p* < 1.8 × 10^–7^), “positive regulation of cold-induced thermogenesis” (*p* < 2.0 × 10^–7^), “response to capsazepine” (*p* < 3.4 × 10^–6^), “adaptive thermogenesis” (*p* < 3.4 × 10^–6^ “lipid metabolic process” (*p* < 1.4 × 10^–4^) and “calcium ion import across plasma membrane” (*p* < 2.3 × 10^–3^). Interestingly, in 3T3-L1 cells 1CP at 8 days, 1CP and 1CPNE at 4 days accounted for the highest percentage of annotated proteins in “brown fat cell differentiation,” “response to cold,” “positive regulation of cold-induced thermogenesis,” and “adaptive thermogenesis.” The highest percentage of annotated proteins of “response to capsazepine” and “calcium ion import across plasma membrane” was recorded by 1CP at 4 days.

In X9 cells, about 70% of annotated proteins involved “brown fat cell differentiation,” “positive regulation of cold-induced thermogenesis” in cells treated with 1CPNE, 0.1CPNE and NE at 4 days, followed by NE at 8 days. The BPs “calcium ion import across plasma membrane” and “response to capsazepine” displayed the lowest protein percentage in all treatments especially in NE-treated cells.

### Principal Component Analysis

**FIGURE 5:**
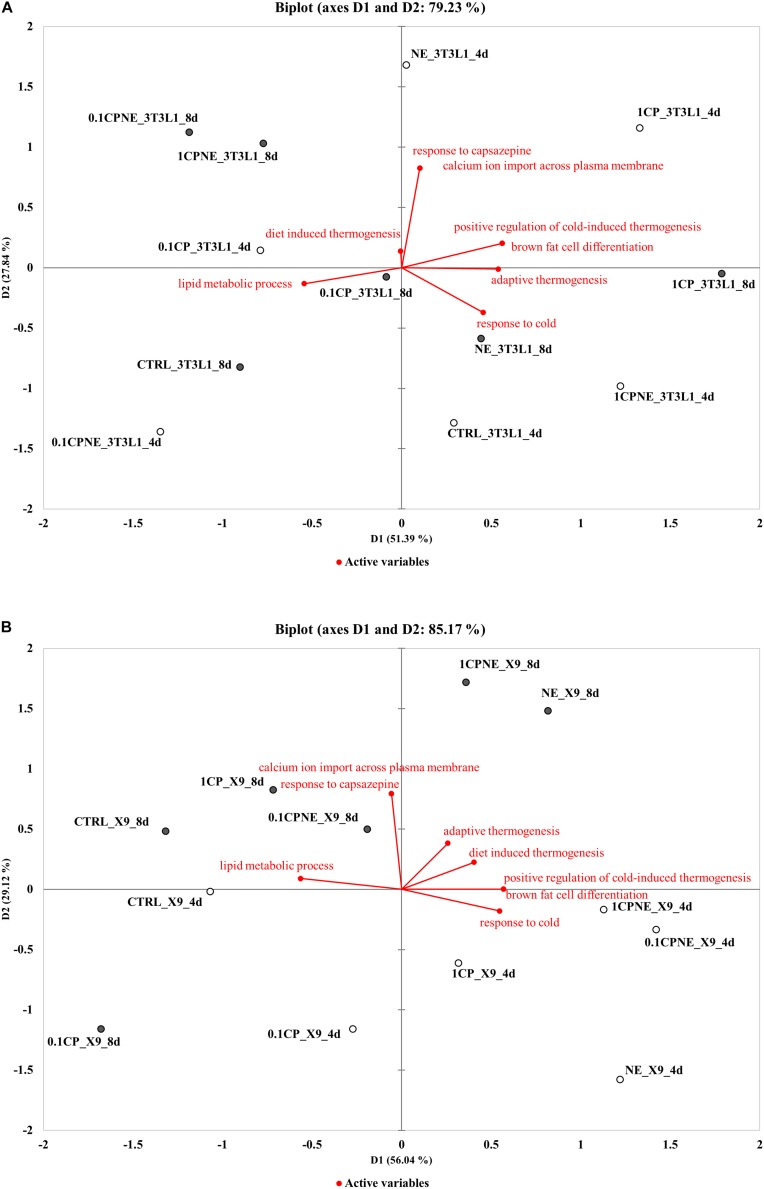
Distance biplots of principal component analysis (PCA) to summarize and visualize the information of the data set containing the treatments (points, active observations) and biological process BPs (vectors, active variables) in **(A)** 3T3-L1 cells at 4 and 8 days and in **(B)** X9 cells at 4 and 8 days.

To better understand the relation between BPs (active variables) and treatments (active observations) in 3T3-L1 cells and in X9 cells at 4 and 8 days, PCA analysis was performed and distance biplots were displayed (Figure 5).

Figure 5A plotted the result of PCA for 3T3-L1 cells. The first two principal components (D1, D2) explained 51.4 and 27.8%, respectively (total variability 79.23%) of the total observed variances of BPs and the individual data were clustered according to treatments and times of differentiation on the first principal component (D1). In this cell line a positive correlation was observed between “calcium ion import across plasma membrane,” “response to capsazepine,” “positive regulation of cold-induced thermogenesis,” and “brown fat cell differentiation.” These BPs were not correlated with “response to cold,” “adaptive thermogenesis,” and “diet induced thermogenesis.” Capsaicin-treated samples 1CPNE at 4 days and 1CP at 8 days were closely related to these BPs and were negatively correlated to “lipid metabolic process.” It is highly significant the position of these active observations which stand completely by themselves and separate from the rest of the fields. This is due to the high values of their percentage of annotated proteins included in active variables (Supplementary Table S5).

Figure 5B plotted the PCA results for X9 cell samples. The first two principal components (D1, D2) explained 56.0% and 29.1%, respectively (total variance 85.17%), of the total observed variances and they clustered treatments between the times of differentiation. Active observations (treatments) at 4 and 8 days were clearly distinguishable in the biplot, grouping with respect to the first principal component (D1) except 0.1CP at 8 days. The BPs “response to cold,” “positive regulation of cold-induced thermogenesis,” and “brown fat cell differentiation” were positively correlated and close to NE, 1CPNE and 0.1CPNE at 4 days observations, which displayed the highest upregulation of browning markers (Table 3) and the highest percentage of protein annotation (Supplementary Table S5). These BPs and the active observations were not correlated with “adaptive thermogenesis” and “diet induced thermogenesis.” BPs showed a negative correlation with “response to capsazepine,” “calcium ion import across plasma membrane” and “lipid metabolic process,” and with the active observations at 8 days.

### Immunofluorescence Analysis of PLIN1, UCP1, and TRPV1 on 3T3-L1 Cells

**FIGURE 6:**
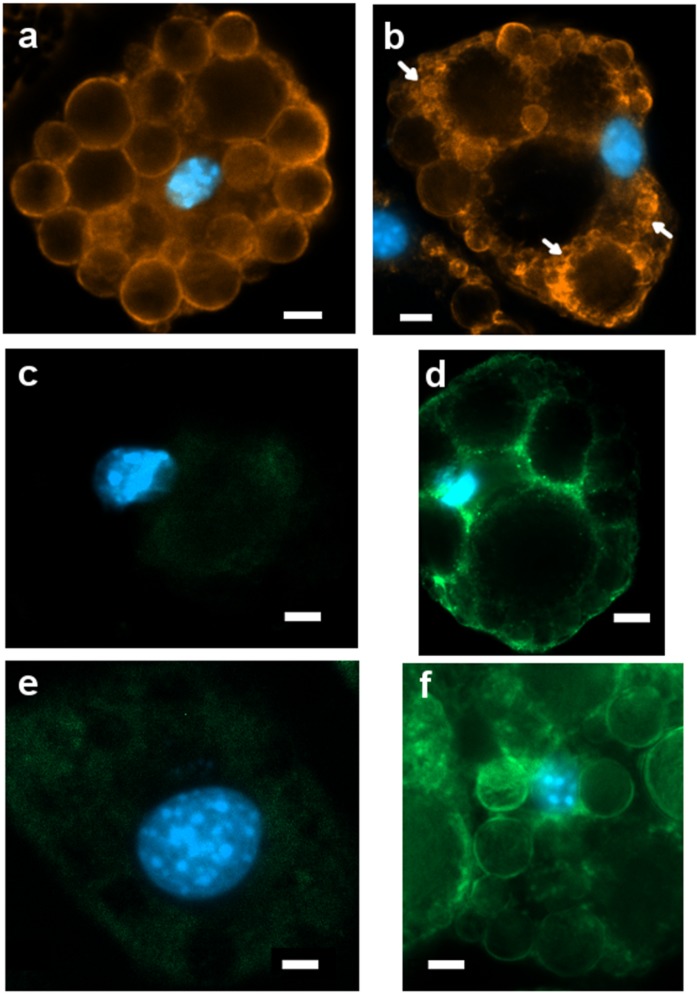
Representative images (ROI) of immunoreactivity in CTRL and 1CP-treated 3T3-L1 cells after 8 days of differentiation (8d). **(a)** PLIN1 positivity along the large LDs in CTRL cells; **(b)** 1CP-treated cells showed PLIN1 immunoreactivity observed along the small and large LDs **(c)** no UCP1 immunoreaction was observed in CTRL cells **(d)** granular UCP1 cytoplasmic positivity in 1CP- treated cells; **(e)** no TRPV1 immunoreaction was observed in CTRL cells **(f)** 1CP-treated cells showed membrane and cytoplasmic TRPV1 immunostaining. Nuclear staining DAPI. Images are representative of n. 5 biological replicates. Scale bars: 5 μm.

In order to deeper investigate the effect of 1CP treatment in 3T3-L1 on protein expression, the immunofluorescence of PLIN1, UCP1, and TRPV1 at 8 days is shown in Figure 6. No reaction was observed in controls performed for each developmental stage by substituting the primary antibodies with PBST blocking solution (data not shown).

PLIN1 showed positive staining in cells, being localized around large LDs and smaller ones in close proximity to them (Figure 6a).

Punctate and disperse cellular staining identified UCP1 expression at 8 days along the circular cytoplasmic rim surrounding nuclei and individual LDs (Figure 6b). TRPV1 was evident along the membranes, although a weak staining was also observed in cytoplasm (Figure 6c).

### Flow of Intracellular Calcium in 3T3-L1 Cells

**FIGURE 7:**
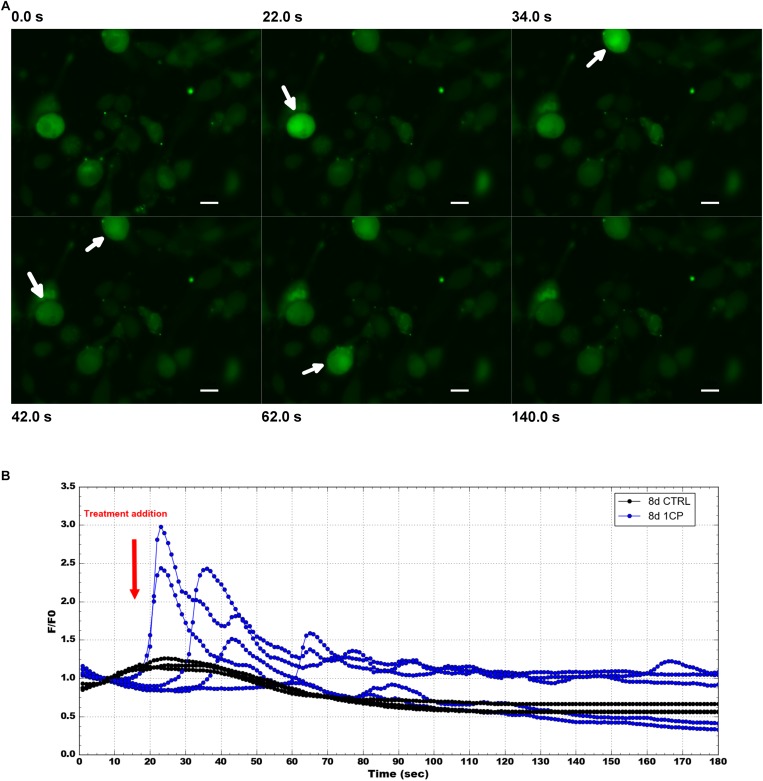
Intracellular Ca^2 +^ imaging and its fluorescence intensity changes on 3T3-L1 cells after 8 days of differentiation. **(A)** Selected images from Fluo-8 AM staining at different times before and after 1 μM capsaicin (1CP) or negative vehicle addition (CTRL) (at 12 s) Scale bars: 20 μm. **(B)** Plotted intracellular Ca^2+^ pulses expressed as the maximum fluorescence signal relative to its starting signal F/F0 and after background subtraction. Blue lines indicate fluorescence intensity after 1CP addition; black lines indicate fluorescence intensity after negative vehicle addition.

To confirm that the physiological effect in 1CP-treated 3T3-L1 cells was achieved by TRPV1 channel activation, the levels of intracellular Ca^2+^ were measured before and after 1CP treatment (Figure 7). *In vivo* imaging of Fluo-8 AM-treated cells showed a ubiquitous localization of internalized Ca^2+^ in adipocytes. The baseline green fluorescence produced bright pulses after capsaicin administration during imaging, strenghtening the observation that capsaicin treatment on 3T3-L1 cells activates TRPV1 receptors, producing an acute Ca^2+^ influx (Figure 7A). Interestingly, maximum fluorescence pulses were produced early after capsaicin addition, within 20 and 42 s, and ended approximately within 120 s as shown by Figure 7B and Supplementary Movie S1. No indicative fluorescence pulses were observed on cells treated with vehicle control or in X9 cells (data not shown).

## Discussion

This study aims to present the effects of different capsaicin treatments on 3T3-L1 and X9 cells, two metabolically distinct murine models, which differ in their functional role and browning response (White and Tchoukalova, 2014). X9 cells are composed of beige adipocytes, isolated from the subcutaneous inguinal adipose depot and display a more powerful response following β_3_-adrenergic stimulation (Wu et al., 2012). While 3T3-L1 cells are an accurate model of WAT depots with visceral features, X9 can represent a subcutaneous pad, more prone to undergo browning. Although the effects of β_3_-adrenergic stimulation appeared within few hours (Barneda et al., 2013), the treatment with NE was chosen to last as the capsaicin treatments (1 or 5 days) to compare the effects of the combination of NE and capsaicin at the same time.

Different functions of white and brown adipose tissues are reflected by LD morphology (Colitti et al., 2018), so the different response to browning of 3T3-L1 and X9 cells under capsaicin and NE treatment can be appreciated from the LD dynamic analysis.

Overall, in 3T3-L1 cells, NE and 1CPNE resulted the most effective treatment considering the LDs measurements. In terms of LDs area surface and MFD, NE treatment and the addition of capsaicin to NE (1CPNE and 0.1CPNE) were effective on 3T3-L1 cells. These results agreed with gene expression analysis recorded with 1CPNE and 0.1CPNE treatments after 4 days of differentiation (Table 2). According to Barneda et al. (2013), 3T3-L1 cells experiencing a β_*3*_-adrenergic stimulation showed a LD size reduction due to lipolysis and a subsequent enlargement of newly formed LDs by a futile cycle of triglyceride hydrolysis and re-synthesis. The IOD value should be carefully considered, since the increase in triglyceride accumulation, evidenced by IOD value (Boschi et al., 2019) in all treatments and control, could be due to a *de novo* synthesis or by a triglyceride trafficking between LDs promoted by the CIDE proteins (Barneda et al., 2015).

Gene expression analysis performed on 3T3-L1 cells highlighted the administration of capsaicin 1 μM as the most powerful treatment on this cellular model, which displayed its greatest effects after 8 days of differentiation. 1CP-treated 3T3-L1 cells at 8 days promoted a significant expression of all brown markers as well as of *Trpv1*, indicating that exposure to capsaicin induced an up-regulation of its receptor. This observation has been confirmed by the detection of the intracellular flux of Ca^2+^ caused by TRPV1 activation. Interestingly, in X9 cells, intracellular flux of Ca^2+^ was not detected. It is known that in BAT TRPV1 activation leads to the SIRT1-mediated deacetylation of PPARγ and PRDM16, facilitating their interaction (Baskaran et al., 2017; Krishnan et al., 2019). Present results are coherent with the observation performed by Baboota et al. (2014), who stated that a 1 μM dose of capsaicin upregulates several thermogenic genes. Capsaicin 1 μM, increased the expression of *Ucp1*, *Tbx1*, *Prdm16*, *Ppargc1a* to trigger a beige phenotype. This was further confirmed by the immunostaining, which evidenced a positive immunoreaction of UCP1 in the cytoplasm of 1CP-treated cells at 8 days and corroborated by the expression of TRPV1 receptor. PLIN1 positivity was observed along small droplets surrounding the biggest ones during their dynamic rearrangement.

No other treatments administered to cultured 3T3-L1 cells produced any significant variation of the expression profile. Notably, NE-treated cells showed the lowest relative fold change values at both time points (Table 2), maybe due to the long NE exposure. In fact, rat epididymal adipose tissue minces exposed to various concentrations of catecholamine showed the highest expression of *Ppargc1a* after 2 h of treatment (Sutherland et al., 2009). On the other hand, it was demonstrated that chronic, but not acute exposure to catecholamine led 3T3-L1 adipocytes toward a brown adipocyte phenotype (Morrison and McGee, 2015). Possibly, the NE induction on gene expression was measured over time and may have induced a negative feedback on the cells (Higareda-Almaraz et al., 2018), thus inhibiting the typical brown-like gene expression profile. However, NE and 1CPNE treatments promoted LDs size reduction possibly activating a futile cycle of triglyceride hydrolysis (and re-synthesis). The up-regulation of *Cidea* relatively to *Cidec*, which did not show any significantly modulation among treatments at 4 days, could be related to the lack of LD fusion. In fact, while CIDEC is crucial for unilocular LD formation, CIDEA alone is not able to maintain the unilocular phenotype (Wu et al., 2014) and its mRNA expression increased during the cold-induced process independently of *Ucp1* expression (Barneda et al., 2013). Moreover, the over-expression of *Plin1* with a corresponding expression of *Cidec* at 8 days, facilitated the LDs enlargement in 1CP-treated 3T3-L1 cells, while CIDEA had a central role in packaging newly synthesized triglyceride in LDs for subsequent lipolysis and fatty acid oxidation useful in thermogenic cells. However, Nishimoto and Tamori (2017) claimed that FSP27β, a novel isoform of CIDEC and abundantly expressed in BAT, is crucial in inhibiting the homodimerization of CIDEA and therefore in the formation of small multilocular LD.

To gain more insight into how capsaicin and NE regulate browning, we performed biological process enrichment analysis against UniProt mouse datasets. In this context, biological process involved in browning and capsaicin treatment were particularly enriched in 3T3-L1 cells at 8 days. The distance biplot of PCA highlighted these associations and, interestingly, 1CPNE and 1CP treatments were related to cold response and were opposite to lipid metabolism. This could suggest the efficacy of prolonged capsaicin treatments on 3T3-L1 cells, while adrenergic treatment was active after shorter stimulation.

The treatments that most affected the LDs dimensions in X9 cells were NE, 0.1CPNE and 1CPNE at both time points. Studies have demonstrated that lipogenesis and lipolysis are coupled in adipose tissue during chronic adrenergic stimulation (Mottillo et al., 2014). The significant decline in triglyceride content could account for the lipolysis, which is an essential prerequisite for UCP1 activation in brown and brite adipocytes (Braun et al., 2018) and in promoting energy dissipation in beige adipocytes independently of UCP1-mediated adaptive thermogenesis (Sepa-Kishi and Ceddia, 2018).

In terms of gene expression, browning markers were up-regulated by NE treatment in X9 cells, while capsaicin doses did not trigger a comparable variation of expression profile. This was confirmed by the fact that *Trpv1*, the membrane receptor for capsaicin, did not show a significant modulation at both time points. This is consistent with the proposed mechanism by which orally administered capsinoids bind to TRPV1 in the gut of mice and human activating a central signal through vagal nerves subsequently transmitted to inguinal WAT depots by the β_2_-adrenoceptor (Snitker et al., 2009; Yoneshiro et al., 2013; Ohyama et al., 2016). Indeed, *Adrb3* was significantly up-regulated by NE at 4 days, activating the expression of browning marker genes. The combination of NE with capsaicin doses produced a significantly lower regulation of brown and brite markers, also in comparison to CTRL cells and to capsaicin treatments.

The distance biplot of PCA of X9 cells, obtained by enrichment analysis, depicted that BPs were involved in browning and response to cold and were associated to a cluster of treatments with NE (NE, 1CPNE, 0.1CPNE) at 4 days. They were negatively related to BPs involved in capsaicin response and lipid metabolism at 8 days. The response to NE observed in X9 cells mimed the cold mediated thermogenesis that stimulates sympathetic efferent (Beiroa et al., 2014), without involving TRPV1 induction, as observed by the lack of Ca^2+^ influx. Instead, in 3T3-L1 cells the observed positive response to browning at 8 days could be directly mediated by the TRPV1.

In terms of LDs dynamic on both cellular models and times of exposure, treatments with NE alone or in association with capsaicin produced a reduction of LDs area surface and size. It should be considered that LDs measurements were done on single cells snapshots in which the LDs remodeling cycle cannot be appreciated. For instance, in capsaicin-treated cells the larger size of LDs could be due to lipogenesis or lipid transference, while the reduction of LDs dimensions in NE-treated cells could be caused by the restart of lipolysis after LD enlargement process during futile cycle.

Present results achieved that *in vitro* chronic capsaicin treatment induced a brite phenotype in 3T3-L1 cells by direct stimulation of TRPV1 receptor. Capsaicin alone did not promote a browning effect on X9 cells, but beige adipocytes were activated, through ADRB3 receptor, only by NE or – to a lesser extent – by NE in association with capsaicin.

## Data Availability Statement

All the datasets for this study are available on request to the corresponding author.

## Author Contributions

TM and MC designed the study. TM performed the experiments and collected data. FB performed statistical analyses and assisted with data analysis. MC collected data measurements, prepared the figures and drafted the manuscript. All authors contributed in interpretation of results and in writing the manuscript.

## Conflict of Interest

The authors declare that the research was conducted in the absence of any commercial or financial relationships that could be construed as a potential conflict of interest.

## Footnotes

1 http://dev.mri.cnrs.fr/projects/imagej-macros/wiki/Lipid Droplets_Tool

2 http://rsb.info.nih.gov/ij/

3 http://imagej.nih.gov/ij/

## Supplementary Material

FIGURE S1 Modulation of MTT metabolism by treatments on 3T3-L1 cells **(A)** and X9 cells **(B)**. Differentiated adipocytes were incubated with different treatments. Values are reported as mean ± standard deviation (SD) of three independent experiments. CTRL = vehicle negative control; NE = 10 μM norepinephrine; 0.1CP = 0.1 μM capsaicin; 0.1CPNE = 0.1 μM capsaicin plus 10 μM norepinephrine; 1CP = 1 μM capsaicin; 1CPNE = 1 μM capsaicin plus 10 μM norepinephrine. 4d = at 4 days of differentiation; 8d = at 8 days of differentiation.

TABLE S1 Lipid droplets measurements in 3T3-L1 cells after 4 (4d) days of differentiation and treated with different doses of compound. Total area surface (μm^2^), MFD, Maximum Feret Diameter (μm); IOD = Intensity Optical Density (dimensionless). CTRL = vehicle negative control; NE = 10 μM norepinephrine; 0.1CP = 0.1 μM capsaicin; 0.1CPNE = 0.1 μM capsaicin plus 10 μM norepinephrine; 1CP = 1 μM capsaicin; 1CPNE = 1 μM capsaicin plus 10 μM norepinephrine. The data are shown as the mean ± standard deviation (SD). Significant pairwise comparisons are indicated in bold.

TABLE S2 Lipid droplets measurements in 3T3-L1 cells after 8 (8d) days of differentiation and treated with different doses of compound. Total area surface (μm^2^), MFD, Maximum Feret Diameter (μm); IOD = Intensity Optical Density (dimensionless). CTRL = vehicle negative control; NE = 10 μM norepinephrine; 0.1CP = 0.1 μM capsaicin; 0.1CPNE = 0.1 μM capsaicin plus 10 μM norepinephrine; 1CP = 1 μM capsaicin; 1CPNE = 1 μM capsaicin plus 10 μM norepinephrine. The data are shown as the mean ± standard deviation (SD). Significant pairwise comparisons are indicated in bold.

TABLE S3 Lipid droplets measurements in X9 cells after 4 (4d) days of differentiation and treated with different doses of compound. Total area surface (μm^2^), MFD, Maximum Feret Diameter (μm); IOD = Intensity Optical Density (dimensionless). CTRL = vehicle negative control; NE = 10 μM norepinephrine; 0.1CP = 0.1 μM capsaicin; 0.1CPNE = 0.1 μM capsaicin plus 10 μM norepinephrine; 1CP = 1 μM capsaicin; 1CPNE = 1 μM capsaicin plus 10 μM norepinephrine. The data are shown as the mean ± standard deviation (SD). Significant pairwise comparisons are indicated in bold.

TABLE S4 Lipid droplets measurements in X9 cells after 8 (8d) days of differentiation and treated with different doses of compound. Total area surface (μm^2^), MFD, Maximum Feret Diameter (μm); IOD = Intensity Optical Density (dimensionless). CTRL = vehicle negative control; NE = 10 μM norepinephrine; 0.1CP = 0.1 μM capsaicin; 0.1CPNE = 0.1 μM capsaicin plus 10 μM norepinephrine; 1CP = 1 μM capsaicin; 1CPNE = 1 μM capsaicin plus 10 μM norepinephrine. The data are shown as the mean ± standard deviation (SD). Significant pairwise comparisons are indicated in bold.

TABLE S5 Enriched analysis of selected significant biological process performed by Funrich finding tool showing the percentage of annoated proteins in 3T3-L1 and X9 cell according to treatments and times. CTRL = vehicle negative control; NE = 10 μM norepinephrine; 0.1CP = 0.1 μM capsaicin; 0.1CPNE = 0.1 μM capsaicin plus 10 μM norepinephrine; 1CP = 1 μM capsaicin; 1CPNE = 1 μM capsaicin plus 10 μM norepinephrine. 4d = at 4 days of differentiation; 8d = at 8 days of differentiation.

MOVIE S1 3T3-L1 cells showing Ca^2+^ transients after capsaicin addition. Maximum fluorescence pulses were recorded at 20 and 42 s.
