# Supplementary figures and images for: Comparison of the Effects of Browning-Inducing Capsaicin on Two Murine Adipocyte Models
Source: Front Physiol. 2019 Nov 5;10:1380. doi: 10.3389/fphys.2019.01380 (PMC6848400; doi:10.3389/fphys.2019.01380)

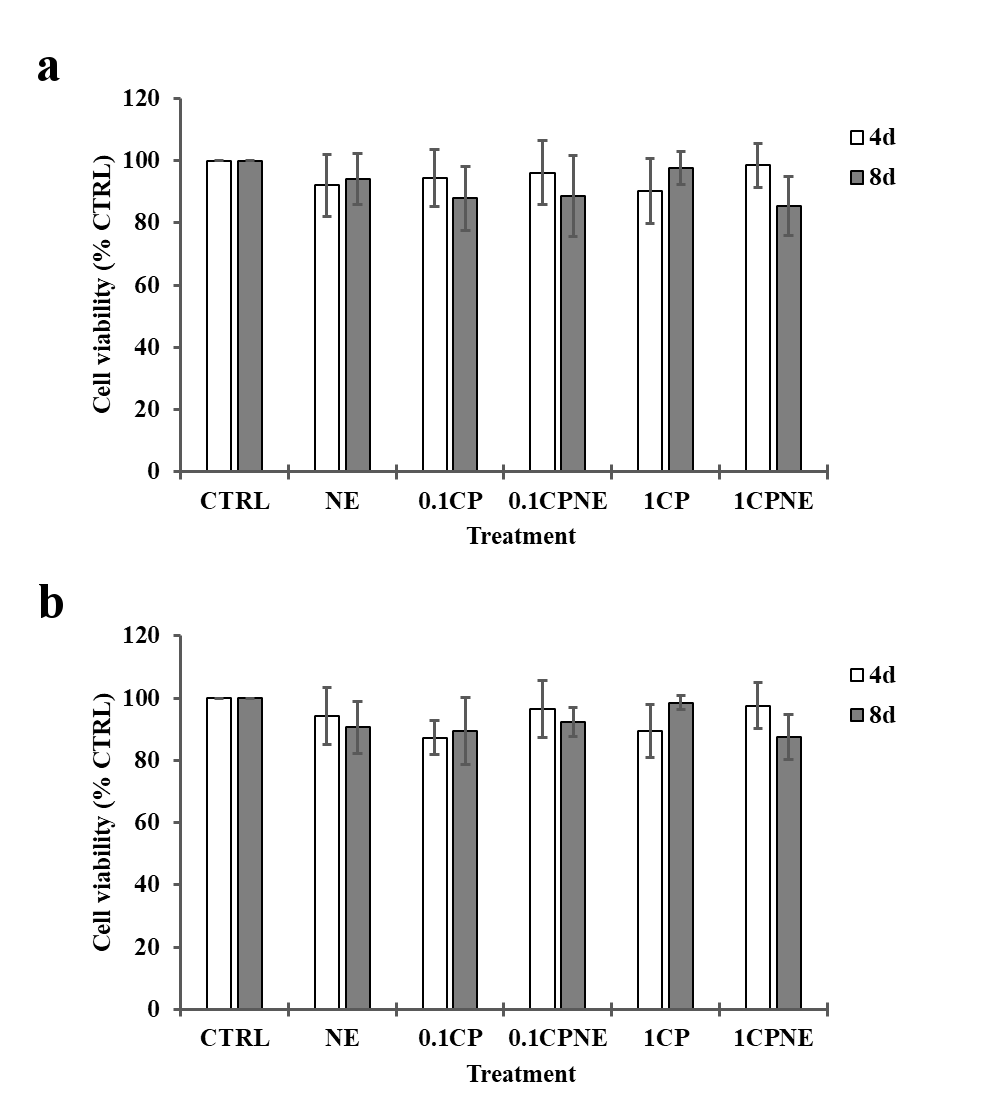

Supplement: FIGURE S1 — Modulation of MTT metabolism by treatments on 3T3-L1 cells (A) and X9 cells (B). Differentiated adipocytes were incubated with different treatments. Values are reported as mean ± standard deviation (SD) of three independent experiments. CTRL = vehicle negative control; NE = 10 μM norepinephrine; 0.1CP = 0.1 μM capsaicin; 0.1CPNE = 0.1 μM capsaicin plus 10 μM norepinephrine; 1CP = 1 μM capsaicin; 1CPNE = 1 μM capsaicin plus 10 μM norepinephrine. 4d = at 4 days of differentiation; 8d = at 8 days of differentiation. [file Image_1.TIF]
